# Newly fabricated zinc oxide nanoparticles loaded materials for therapeutic nano delivery in experimental cryptosporidiosis

**DOI:** 10.1038/s41598-023-46260-3

**Published:** 2023-11-10

**Authors:** Doaa A. Hamdy, Mousa A. M. Ismail, Hala M. El-Askary, Heba Abdel-Tawab, Marwa M. Ahmed, Fatma M. Fouad, Fatma Mohamed

**Affiliations:** 1 Department of Medical Parasitology, College of Medicine, Beni-Suef University, Beni-Suef, Egypt; 2 Department of Medical Parasitology, College of Medicine, Cairo University, Giza, Egypt; 3 Department of Zoology, Faculty of Science, Beni-Suef University, Beni-Suef, Egypt; 4 Department of Pathology, College of Medicine, Beni-Suef University, Beni-Suef, Egypt; 5 Nanophotonics and Applications (NPA) Lab, Faculty of Science, Beni-Suef University, Beni-Suef, 62514 Egypt; 6 Materials Science Lab, Chemistry Department, Faculty of Science, Beni-Suef University, Beni-Suef, 62511 Egypt

**Keywords:** Zoology, Chemistry, Materials science

## Abstract

Cryptosporidiosis is a global health problem that threatens the lives of immunocompromised patients. This study targets to fabricate and investigate the efficiency of zinc oxide nanoparticles (ZnO-NPs), nitazoxanide (NTZ)-loaded ZnO-NPs, and *Allium sativum *(*A. sativum*)*-loaded* ZnO-NPs in treating cryptosporidiosis. Further FTIR, SEM, XRD, and zeta analysis were used for the characterization of ZnO-NPs and loaded materials. The morphology of loaded materials for ZnO-NPs changed into wrapped layers and well-distributed homogenous particles, which had a direct effect on the oocyst wall. The charge surface of all particles had a negative sign, which indicated well distribution into the parasite matrix. For anti-cryptosporidiosis efficiency, thirty immunosuppressed *Cryptosporidium parvum-infected* mice, classified into six groups, were sacrificed on the 21st day after infection with an evaluation of parasitological, histopathological, and oxidative markers. It was detected that the highest reduction percent of *Cryptosporidium* oocyst shedding was (81.5%) in NTZ, followed by (71.1%) in *A. sativum-loaded* ZnO-NPs-treated groups. Also, treatment with *A. sativum* and NTZ-loaded ZnO-NPs revealed remarkable amelioration of the intestinal, hepatic, and pulmonary histopathological lesions. Furthermore, they significantly produced an increase in GSH values and improved the changes in NO and MDA levels. In conclusion, this study is the first to report ZnO-NPs as an effective therapy for treating cryptosporidiosis, especially when combined with other treatments that enhance their antioxidant activity. It provides an economical and environment-friendly approach to novel delivery synthesis for antiparasitic applications.

## Introduction

According to predictions made by Kerri et al. and Liu et al.^1, 2^,* Cryptosporidium* is the primary cause of paediatric diarrhoea worldwide. Cryptosporidiosis is transmitted via the faecal-oral route, animal-to-human (zoonotic), and direct human-to-human transmission^3^. It is largely a self-limiting disease in immunocompetent hosts, but it leads to significant morbidity in immunocompromised people. Lack of options for antiparasitic therapies, keeping in mind the management limitations in this category of patients, makes treatment particularly challenging^4, 5^.

Henceforth, the current study aimed to (i) Fabricate and investigate the efficiency of Zinc oxide nanoparticles (ZnO-NPs), Nitazoxanide (NTZ) loaded ZnO-NPs and *Allium sativum *(*A. sativum*) loaded ZnO-NPs in treating cryptosporidiosis. (ii) Evaluate the efficacy of fabricated material through histopathological and parasitological examinations. (iii) Estimate values of some relevant oxidative stress markers.

There are few pharmaceutical treatments for *Cryptosporidium* infection. The only medication that the Food and Drug Administration has approved is NTZ^6^. NTZ reduces oocyst shedding and treats diarrhoea for 3–4 days^7^. Results in HIV-infected individuals are not encouraging, and there is marked debate on the efficacy of all available medications^8^. To reduce the high burden of the disease, it becomes a challenge to find out new alternative treatments for cryptosporidiosis. In addition, new approaches to identify possible therapeutic targets are needed to improve the development of anti-cryptosporidial therapy^9^.

According to recent studies, it is pharmacologically conceivable to treat cryptosporidiosis with new types of antiprotozoal drugs and natural remedies^10^. Nanoparticles have significant potential for the efficient treatment of parasitic illnesses as an emerging medication carrier. Numerous studies have focused on the need to establish the ideal formula for combining nanoparticles with particular anti-protozoal medications to treat cryptosporidiosis^11, 12^.

One of the most widely studied herbal supplements in the field of traditional medicine is garlic (*A. sativum*) and its variable commercial preparations^13^. Its potential uses as a preventive measure and adjuvant treatment are of current interest^14^. *A. Sativum* is an effective drug in the treatment and prevention of atherosclerosis and possesses hypotensive, anti-inflammatory, antioxidant, antifungal, antibacterial, antiviral, anticancer, antithrombotic, and immunomodulatory characteristics as well. Mainly, the therapeutic properties of *A. sativum* are owed to the presence of the most bioactive and potent components, allicin and thiosulfinates (sulphur-containing compounds)^15^*.*

*A.Sativum* has an anti-parasitic effect against many parasites, such as* Taenia taeniaeformis, Hymenolepis diminuta, Hymenolepis microstoma, Fasciola hepatica, Echinostoma caproni,* and *Echinococcus*^16, 17^. Garlic extracts also have anti-protozoal activity against* Blastocystis, Cryptosporidium parvum, Leishmania tropica and major, Giardia lamblia, Entamoeba histolytica, Plasmodium, Trypanosoma,* and *Toxoplasma gondii*^18–25^*.* Antischistosomal activities of *A. sativum* and its efficacy against *Schistosoma mansoni* were suggested in previous studies^26, 27^.

There is a trend for applying nanotechnology in the fields of physics, chemistry, biology, and nanomedicine. Metal oxides have gained amazing attention because of their significant characteristics and diverse applications in materials sciences, physics, and chemistry^28, 29^.

Zinc oxide (ZnO) is used as an antibacterial agent because it has the qualities of Zn, a mineral that is necessary for human health, non-toxic, long-lasting, and powerful even at lower concentrations. Zinc as an essential trace element, plays a vital role in human enzymatic activity. Zinc participates in variable biological activities, such as phagocytosis, replication, gene translation, transcription, cytokine production, and immunoglobulin production^30–33^.

One of the five zinc compounds that are recently commonly believed and registered nowadays as safe by the US Drug and Food Administration is zinc oxide nanoparticles (ZnO-NPs)^34^. ZnO-NPs, with their small particle size, are absorbed by the body more easily. Compared with other metal oxide NPs, ZnO-NPs are cheap and have less toxic properties that exhibit perfect applications in the biomedical field, such as drug delivery, anti-diabetes, anticancer, antibacterial, wound healing, anti-inflammatory, and bioimaging^35^.

Biomedical nanomaterials have recently attracted great interest owing to their significant biological properties and potential applications in medicine. Metal oxide nanoparticles display promising materials for the biomedical field^36^. Due to their biocompatibility, economic advantage, and low toxicity, ZnO is regarded as a good contender in biocompatible applications among these oxides^37, 38^.

## Materials and methods

### Animals and experimental design

The current study was implemented on 30 laboratory-outbred Swiss albino mice of the most widely cost-effective and fully genetically variable known strain (CD1 strain), Each mouse was about 3–5 weeks old and weighing 20–25 gm each. All experimental procedures were carried out in accordance with the internationally valid guidelines for animal experimentation and were approved by the Institutional Animal Care and Use Committee of Beni-Suef University (BSU-IACUC). The study was carried out in accordance with the ARRIVE guidelines. They were used and divided into six groups; each group consists of five mice. The provided animals were from the *Schistosoma* Biological Supply Programme (SBSP) at Theodor Bilharz Research Institute (TBRI). Mice were housed in the animal house of the Faculty of Science at Beni-Suef University with clean wood-chip bedding, well-aerated cages, with food pellets, water, and libitum at a temperature of 24 °C. Their stools were examined by iodine wet mount and modified Ziehl–Neelsen stain^39^ before and after concentration techniques^40^ to exclude the existence of parasites.

(i) All included mice were immunosuppressed and classified into 2 main groups, and each group was subdivided into 3 sub-groups:
(ii) Control groups:

1. Negative control: non-infected non-treated group.
2. Positive control: (*C.parvum*) infected non-treated group.
3. *C.parvum* infected mice receiving NTZ (drug control group).
4. NPs treatment groups:
5. *C.parvum* infected mice receiving ZnO-NPs with dose of 10mg/kg.
6. *C.parvum* infected mice receiving NTZ loaded on ZnO-NPs.
7. * C.parvum* infected mice receiving *A.sativum* loaded on ZnO-NPs.

### Immunosuppression

Immunosuppression was carried out by synthetic dexamethasone (Dexazone) given orally using an esophageal tube at a dose of 0.25 µg/g/day for 15 days before inoculation with *Cryptosporidium* oocysts^41^. Dexazone (dexamethazone) (0.5 mg) was commercially produced and presented by Kahira Pharmaceuticals and Chemical Industries Company, Shoubra-Cairo, Egypt.

### Preparation of *Cryptosporidium* oocysts and mouse inoculation

*Cryptosporidium Parvum* oocysts used for infecting mice were obtained from diarrheal young calves coming to veterinary clinics in the Faculty of Veterinary Medicine at Beni-Suef University. Faecal specimens were collected in clean sterile cups, provided that samples were not mixed with urine or water, and then transferred to the Zoology Department at the Faculty of Science at Beni-Suef University.

Collected stool specimens were exposed to macroscopic examination and then examined microscopically by Modified Ziehl–Neelsen stain (MZN) (cold method) to identify *Cryptosporidium* oocysts^42^. The same volume of 2.5% potassium dichromate (K_2_Cr_2_O_7_) was used to preserve the proved positive samples for *Cryptosporidium* and stored at 4 °C) until used for mouse inoculation^43, 44^. Distilled water was used to wash *Cryptosporidium* oocysts three successful times to get rid of potassium dichromate and centrifuged at 1500×*g* for 10 min just before use^45^.

The calculated infecting dose was done by taking the mean of three counts of MZN-stained oocysts and examining them under the oil immersion lens. Each count was done by taking 50 µl from the stool specimen and counting the oocysts in it, repeating this three times, and then taking their mean. Multiplication by 20 was done to get the result in 1 ml of the specimen^46^. Animals other than the negative control group were infected with *Cryptosporidium* oocysts orally by esophageal intubation; traumatic injury to animal throats was avoided. Nearly each mouse was infected by a dose of 104 Cryptosporidium oocysts^45^.

To make sure of mice infection, stool pellets were brought from different mice groups from the 2nd day post-infection (PI) up to 1 week later, then examined parasitologically by MZN stain (cold method) for detection of *Cryptosporidium* oocysts^42^.

### Drug dosage and administration

All doses of used drugs in this study were calculated in grams according to mice weight (each mouse is approximately 20–25 gm), starting 7 days PI, once daily, and lasting for 5 days later.

NTZ (Nanazoxid) was administered orally using an esophageal tube in suspension form at a dose of 100 mg/kg body weight^47, 48^. Powders of all the drugs loaded on ZnO-NPs were added to distilled water and then sonicated to form a suspension that was administered orally using an esophageal tube. NTZ-loaded ZnO-NPs powder was given in a dose of 100 mg/kg^48^. *A. sativum-*loaded ZnO-NPs were administered at a dose of 500 mg/kg^49^. ZnO-NPs were given at a dose of 10 mg/kg^50^. Experimental animals were scarified on the 21st day of PI. Scarification was done by intraperitoneal anesthesia. An anticoagulant anaesthetic solution (100 units/ml heparin and 500 mg/kg thiopental) was injected intraperitoneally into the mice^51^.

### Preparation of nanoparticles

#### Preparation of ZnO-NPs

Zinc sulphate Zn (So4) 2H2O, sodium hydroxide (NaOH), and trisodium citrate solution were used for the preparation of ZnO-NPs. All chemicals were provided by Sigma Aldrich, ready to use, with no further purification. Both solutions contain 0.2 molar NaOH and 0.1 molar Zn sulfate. 2H2O was dissolved in deionized water. After that, both solutions were poured into one beaker and stirred at 1000 rpm for up to 4 h (at a temperature of 60 ºC). Then, the transparent original solution became milky white. The solution was centrifuged at 4500 rpm for 2 min., resulting in the precipitation of a white product. Later, it was washed with deionized water and then with methanol. Powdered ZnO-NPs were calcinated at 350 ºC for 3 h^52^.

#### Preparation of NTZ and A. sativum-loaded ZnO-NPs

ZnO-NPs (0.05 g) were added to 100 ml of trisodium citrate solution 5.4 mM (Merck, Germany) and stirred for 1 h. When 2 gm of grinded NTZ powdered in an aqueous solution and 2 gm of *A. sativum *powdered in another aqueous solution, ZnO-NPs were added to each solution separately under sonication, stirred overnight at room temperature, and filtrated. The powder was washed twice with distilled water and methanol, then dried^53, 54^.

#### Characterization of fabricated samples

The fabricated samples were characterised by XRD (PANalytical Empyean, Sweden) at 40 kV, 30 mA current, ranging from 5 to 60o scan angle, accelerating voltage, and a scan step of 0.05. To determine the vibration of chemical bonds, Bruker (vertex 70 FTIR Raman) Germany spectrophotometry (serial number 1341) covering a frequency range of 400–4000 cm1 was applied using a potassium bromide disc. The morphology of materials had been assessed through a scanning electron microscope (SEM) (JEOL (JSM-5200), Japan).

For evaluating zeta potential and particle size distribution, the stability of the suspensions was evaluated in water. A Malvern Instruments Ltd., Worcestershire, UK, Zetasizer Nano ZS device with an MPT-2 automatic titrator was used to assess zeta potential in 10 mM NaCl. Due to its ideal ionic strength, this solution is useful for determining zeta potential. The hydrodynamic diameter of the particles in ultrapure water was measured by dynamic light scattering (PCS-Photon Correlation Spectroscopy). A 173° angle was measured between the scattering and the incident He/Ne laser beam. The Contin algorithm's autocorrelation functions were used to generate particle size distributions that were intensity-weighed. The intensity-weighted average hydrodynamic diameter was obtained by doing a second-order cumulant analysis of the correlation function and using the Stokes–Einstein relation while taking the suspension's viscosity into consideration.

### Assessment of drug efficacy

#### Parasitological assessment

On day 21, PI, faecal pellets were weighted, dissolved in formol saline at 10%, and filtered by sterile gauze to obtain a clear film. Then, by micropipette, 50 µl were taken from each specimen and examined parasitologically using MZN stain to count the number of *Cryptosporidium* oocysts^36^. Then, OPG (oocyst per gram of faeces) was calculated^55^.

The % reduction in the OPG count was calculated referring to this equation: %R = 100(C − E)/C.

%R: reductions; C: control group; and E: experimental groups of mice^56^.

#### Histopathological assessment

The small intestinal (ileum) segments, liver, and lung specimens excised from scarified mice were subjected to histopathological preparation. The excised specimens were cut both transversely and longitudinally, fixed in 10% formalin, and oriented on filter paper. Tissues were processed for paraffin embedding after fixation. Histopathological sections of 5 μm thickness were processed from each block and stained with Haematoxylin and Eosin stain (Hx&E) according to Drury and Wallington^57^. Histopathological examination was performed for detection of *Cryptosporidium* oocysts, associated pathological changes, and assessment of drug efficacy after treatment regimen.

#### Oxidative stress marker assessment

From different groups, parts of the ileum were gathered and homogenised in cold buffer (i.e., 100 mM potassium phosphate, pH 7.0, containing 2 mM EDTA) to yield a 50% (w/v) homogenate. Next, the homogenate was centrifuged for 15 min at 4000 rpm.

Determination of Glutathione Reduced (GSH): The principle was producing a yellow compound from the reduction of 5,5′ dithiobis (2-nitrobenzoic acid) (DTNB) with GSH. The GSH concentration is directly proportional to the reduced chromogen. Its absorbance was measured at 405 nm and expressed as mmol/gm tissue following the manufacturer’s instructions (Biodiagnostics, Co.)^58^.

Determination of Nitric Oxide (NO): The principle was the formation of nitrous acid diazotise sulphanilamide in the presence of nitrite and in an acidic medium. *N*-(1-naphthyl)-ethylenediamine was coupled to the product. A bright reddish-purple colour appeared to the resulting azo dye that was measured at 540 nm and expressed as μmol/gm tissue following the manufacturer’s instructions (Biodiagnostics, Co.)^59^.

Determination of Lipid Peroxide (Malondialdehyde [MDA]): The principle was the formation of a thiobarbituric acid reactive product by the reaction of thiobarbituric acid (TBA) with MDA in an acidic medium at a temperature of 95 °C for 30 min. The resultant pink product absorbance was measured at 534 nm and expressed as nmol/gm tissue following the manufacturer’s instructions (Biodiagnostics, Co.)^60^.

### Ethical consideration

All experimental procedure and animal maintenance were approved by the Institutional Animal Care and Use Committee of Beni-Suef University (BSU-IACUC) with approval number (021-127).

## Statistical analysis

All data were quantitatively analyzed by one-way ANOVA. Subsequently, multiple comparisons were done between groups. Results were enumerated as mean ± SEM, and the value of significance was set at *P* ≤ 0.05. GraphPad Prism v.6.0 (GraphPad Software, San Diego, CA, USA) was used to accomplish all calculations.

## Results

### Characterization of the prepared samples

**Figure 1:**
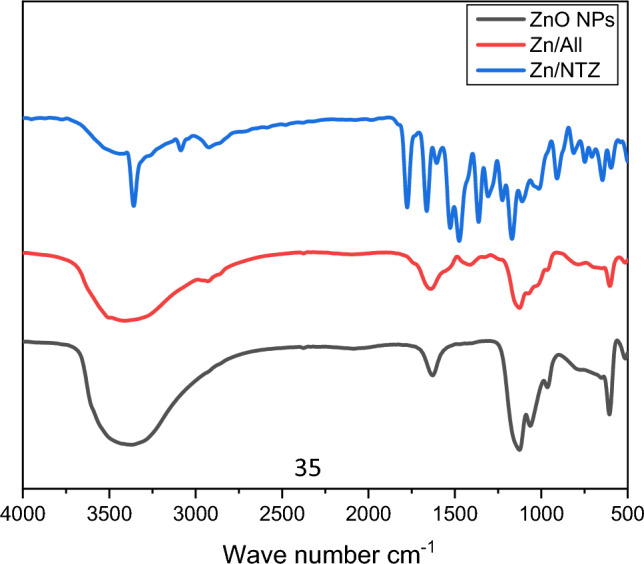
FTIR of ZnO-NPs, (Zn/All) and (Zn/NTZ). The functional groups present in samples were evaluated by FTIR analysis. The FTIR spectra were recorded within the range of 400–4000 cm^−1^.

The existence of diverse functional groups accountable for the production of ZnO-NPs in bacterial biomass was explored using FTIR spectroscopy. FTIR analysis was looked into to determine the chemical functional group of the obtained ZnO, A. sativum-loaded ZnO-NPs (Zn/All), and NTZ-loaded ZnO-NPs (Zn/NTZ). Figure 1 displays FTIR spectra of the loaded samples and Zn-ONPs. The analysis of the FTIR spectrum gave insight into the bimolecular structure carrying various functions that are present in the underlying system. In the case of ZnO, a large absorption peak that was attributable to the adsorbed water molecules emerged at about 3920 cm1. At 1600 cm1, a short, sharp absorption peak was seen, which was attributed to the aromatic bending of the alkene group (C=C). The sharps peak at 1130 cm1 is ascribed to the C-H band's deformation vibration.^61, 62^

While after loading with A. sativum (*Zn/All*)*, the* FTIR spectrum was represented in Fig. 1, there was a red shift after loading. Absorption peaks at 3405 cm^−1^ were corresponding to the presence of flavonoids, non-flavonoids, and saponins in illum^63, 64^.

In addition, peaks appeared at 2920 cm1, which corresponded to the asymmetric stretching of C-H groups in aromatic compounds. While organosulfide compounds such as alliin, allicin, and diallyl disulfide were detected at the peak at 1130 cm1, which was attributed to the S=O group.

Furthermore, in the loading of NTZ on ZnO-NPs (Zn/NTZ), some absorption peaks appeared; a sharp peak appeared at 3357 cm1, which was attributed to N–H stretching vibration; and some new peaks were created as a result of the formation of a complex between the drug and ZnO-NPs. In addition, some absorption peaks shifted to a higher frequency^65^.

**Figure 2:**
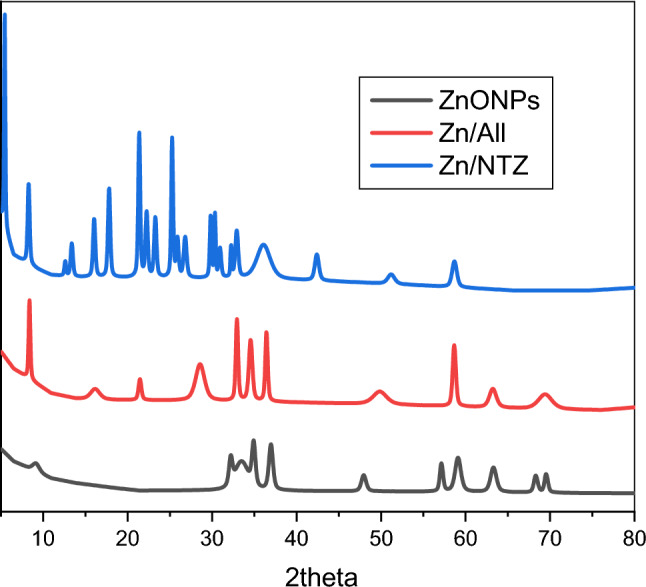
XRD Pattern of ZnO-NPs, (Zn/ All) and (Zn/NTZ) from 10 to 80 theta to show the crystallinity of samples.

The structural properties of these materials were examined through XRD diffraction, as shown in Fig. 2. The XRD pattern of pure ZnO exhibited high crystallinity with no secondary phases, where the presence of (100), (002), and (101) planes matched the hexagonal wurtzite crystal structure of ZnO **.** The structural properties were modified after loading. The position of the diffraction peaks of (Zn/All) changed and shifted due to the formation of a complex between ZnO and *A. sativum*.

To know about the phase identification and nano-crystalline structure of the ZnO and loaded nanoparticles,XRD diffraction must be investigated^66^.The XRD of pure NTZ, physical mixture and solid dispersion is shown in Fig. 2. The XRD spectra of NTZ showed many distinct peaks, indicating that the drug was in highly crystalline form, and the same was also depicted in the case of physical mixtures. But the intensity of these peaks decreased in solid dispersions, exhibiting a decrease in the crystallinity of NTZ^67–71^.

**Figure 3:**
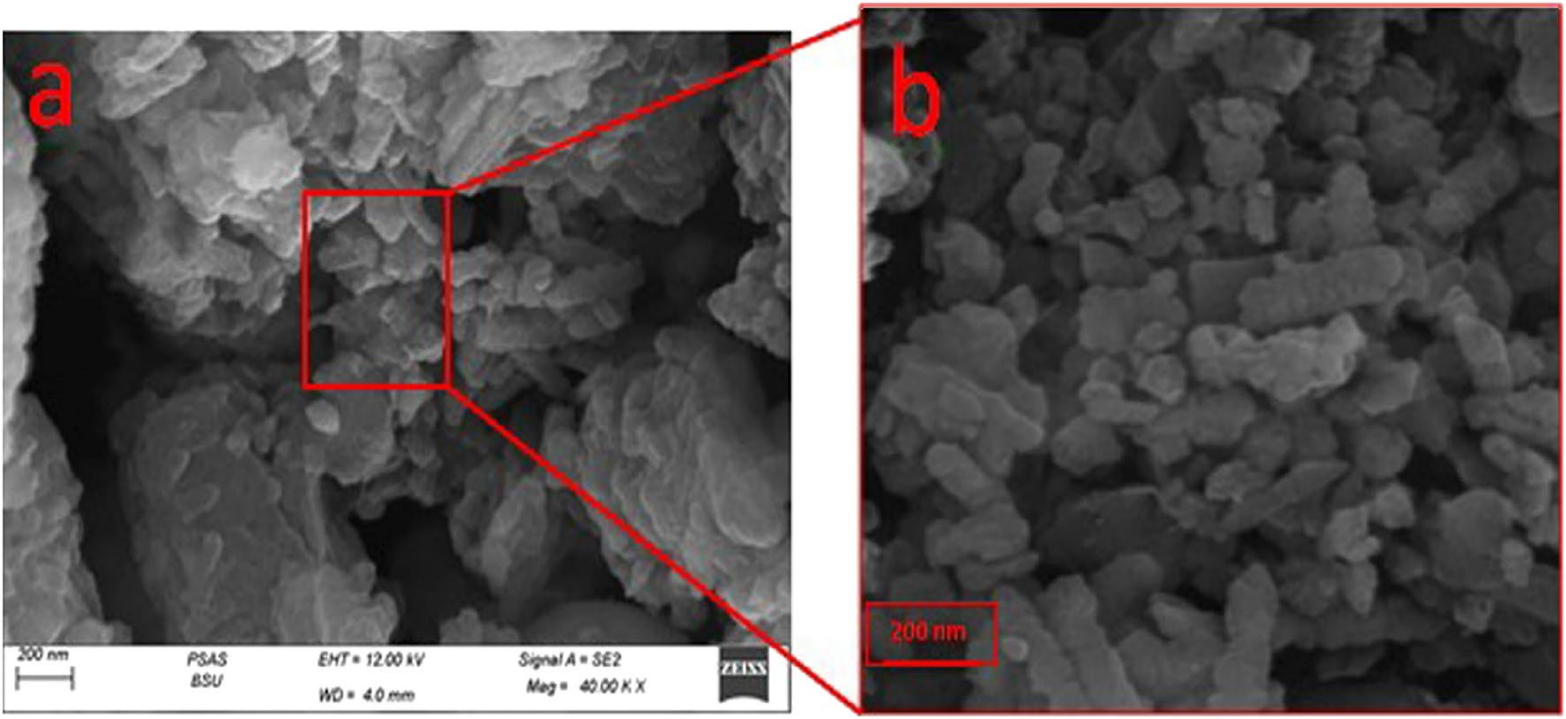
SEM of ZnO-NPs illustrating the surface morphology of sample.

**Figure 4:**
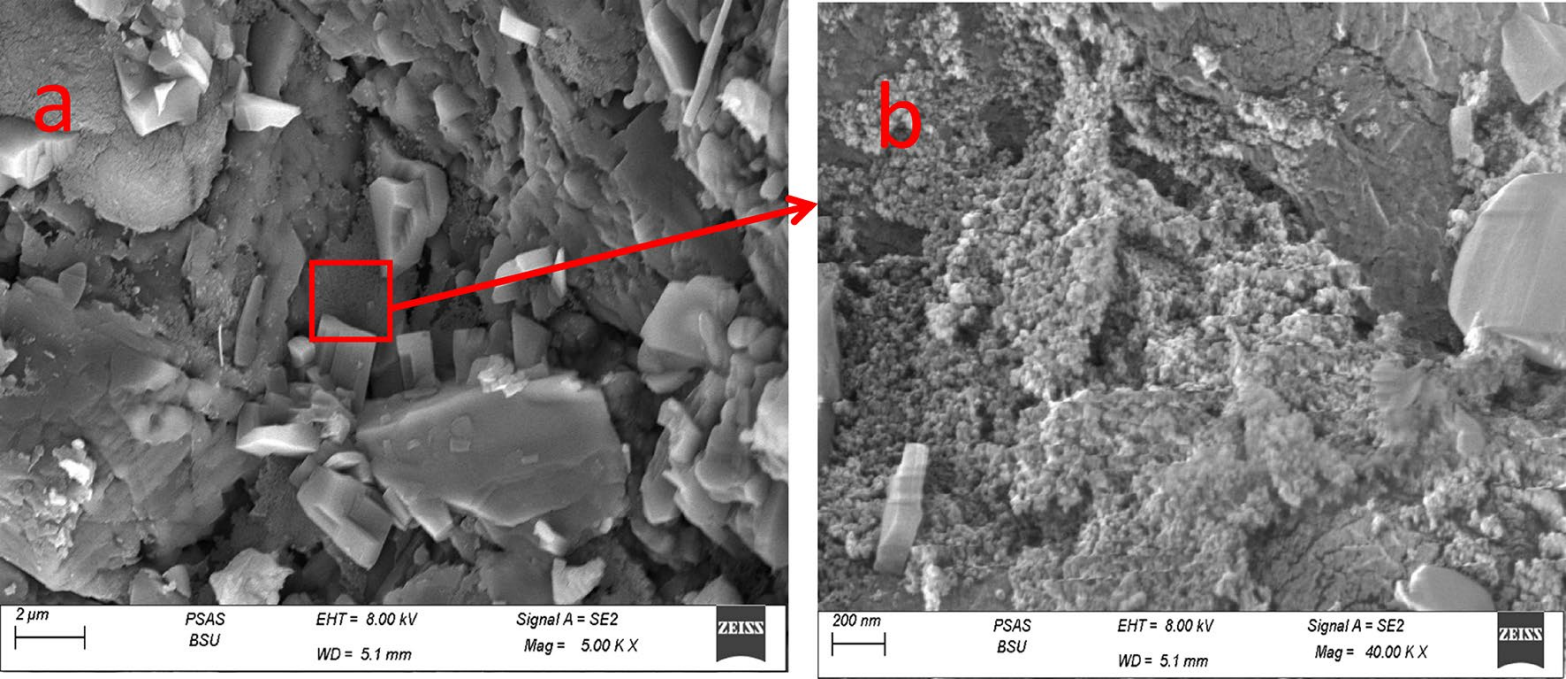
SEM of Zn/All illustrating the surface morphology of sample.

**Figure 5:**
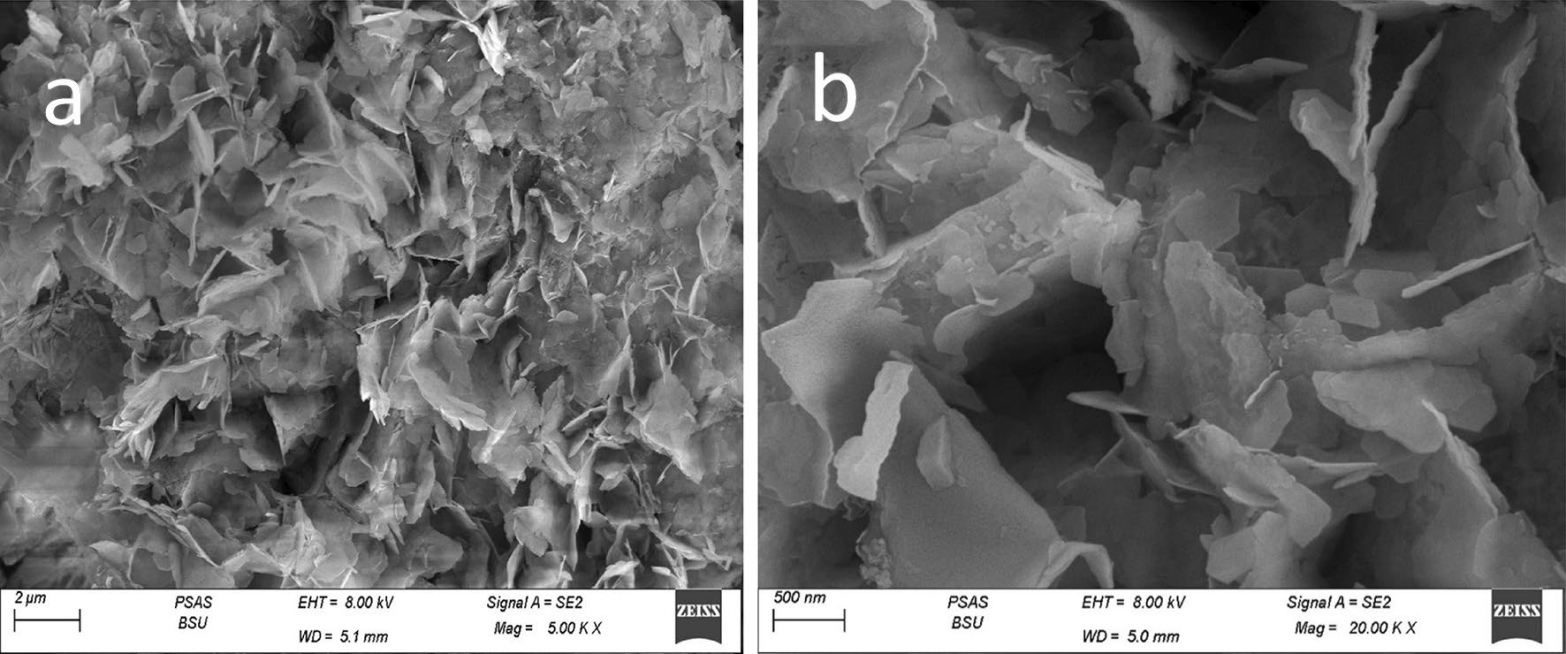
SEM of Zn/NTZ NPs illustrating the surface morphology of sample.

Pure ZnO-NPs were confirmed by SEM to be aggregated into a layered structure, as shown in Fig. 3a. According to Fig. 3b, Zno-NPs emerged as uniform, spherical particles stacked on top of one another. These particles ranged in size from 50 to 65 nm on average. SEM images of (ZN/All) were displayed in Fig. 4 a, b after loading. There seemed to be a lot of granules evenly dispersed across the surface of A. sativum. ZnO is visible as strewn particles covering the entire surface of A. sativum. In the SEM of Zn/NTZ, the composite morphology between the drug and ZnO was fully altered and transformed at Fig. 5 a, b, and the surface showed wrapped layers^72, 73^.

**Figure 6:**
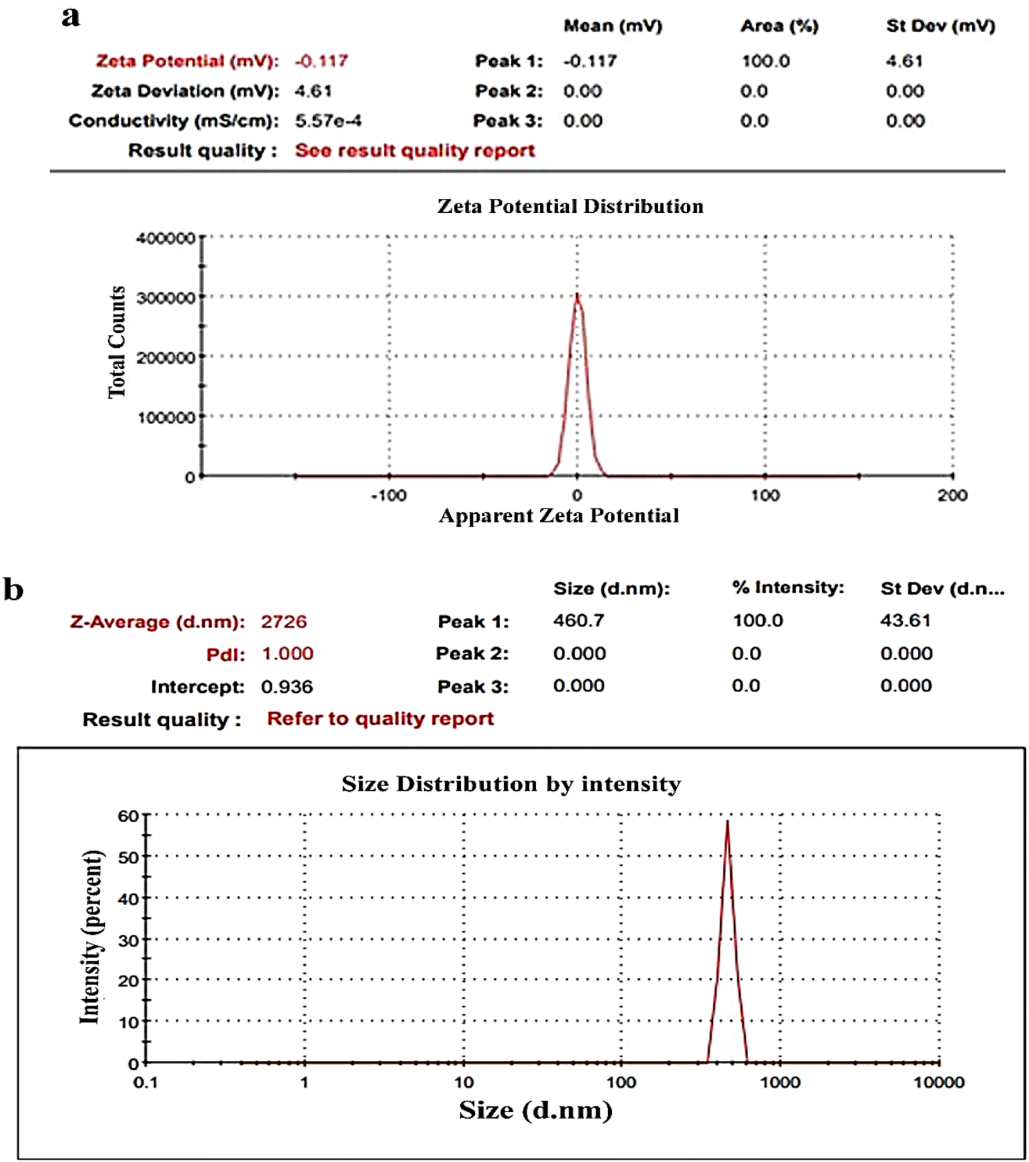
(**a**) ZnO-NPs zeta potential, (**b**) ZnO-NPs particle size distribution.

**Figure 7:**
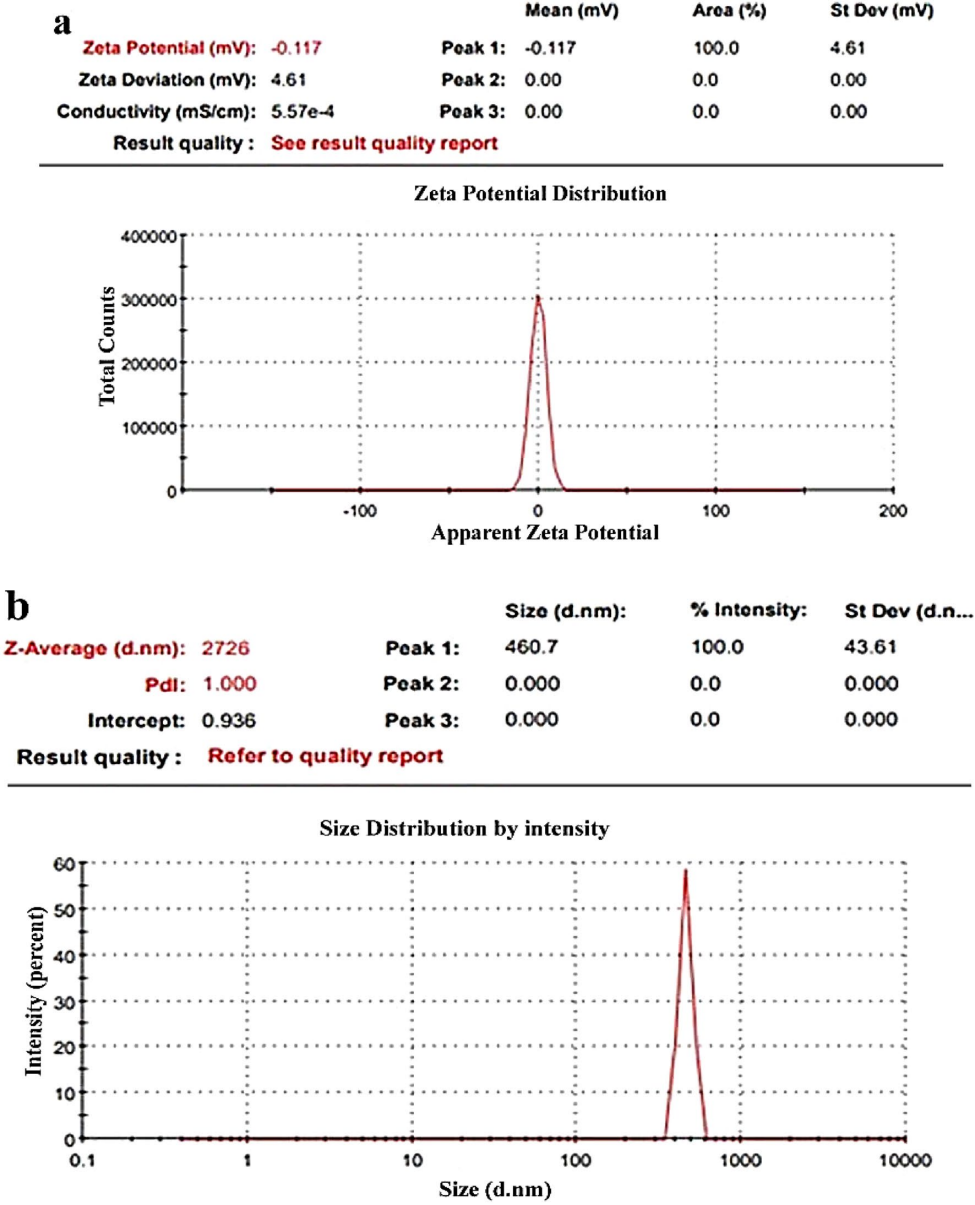
(**a**) Zn/All zeta potential, (**b**) Zn/All particle size distribution.

**Figure 8:**
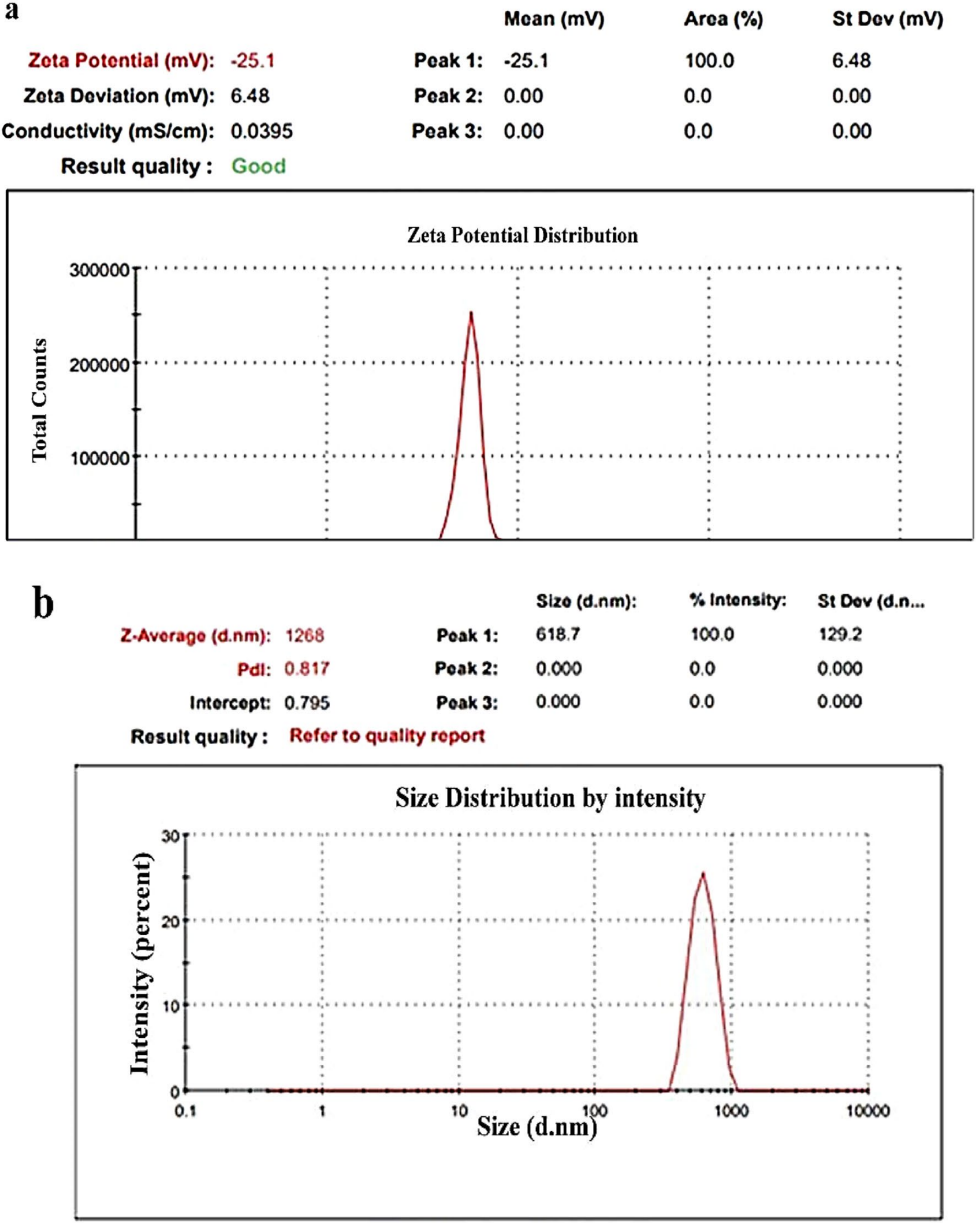
(**a**) Zn/NTZ zeta potential, (**b**) Zn/NTZ particle size distribution.

Zeta potential measurements in Figs. 6, 7, and 8 validated the stability and charge of all samples based on ZnO-NPs. For pure ZnO, Zn/All, and Zn/NTZ, respectively, it was seen that all samples had negative charges (− 12.4 mV, − 25.1 mV, and − 0.11 mV). The stability and charge of the nanoparticles were assessed using zeta potential assays based on their electrophoretic mobility. Zeta potential value was considerably reduced after being loaded with A. sativum and NTZ.As-made ZnO-NPs and samples that had been loaded with A. sativum and NTZ both had hydrodynamic diameters of 535, 1268, and 2726 nm, respectively, as shown in Figs. 6, 7, and 8b^74^.

### Morphology of immunosuppressed murine models

Signs of immunosuppression started to appear on mice after two weeks from drug administrative doses by existence of subcutaneous edema, cutaneous ulcerations and hair loss.

### Effect of NTZ and different NPs treatment regimens on OPG count

**Figure 9:**
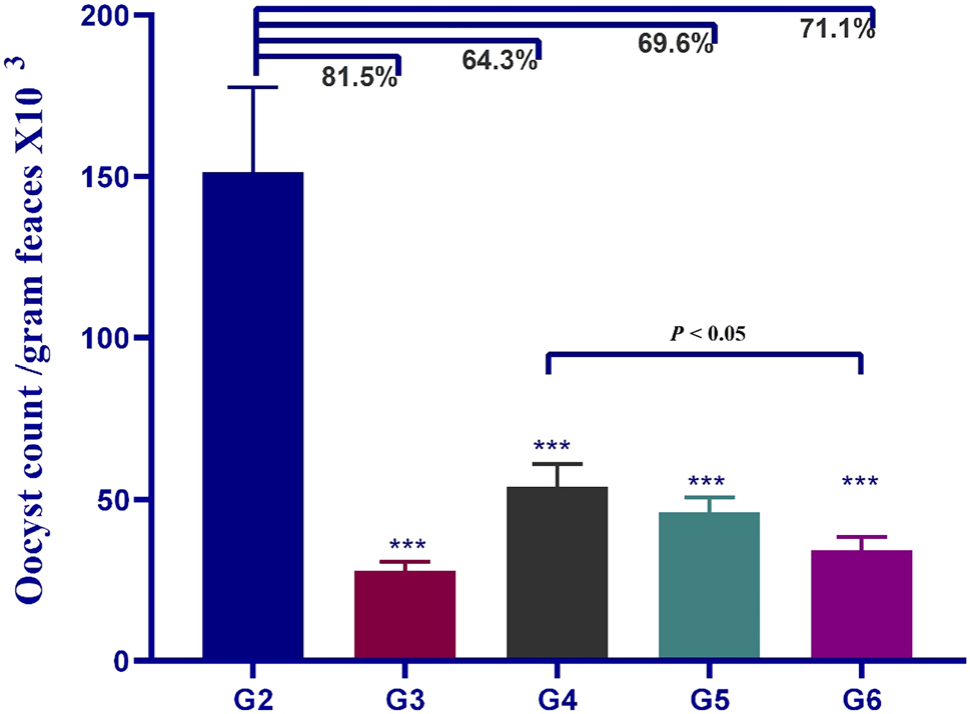
Mean oocyst count among the different groups. G2*.C. parvum* infected non-treated group. G3. *C. parvum* infected mice treated with NTZ. NPs treatment groups: G4. *C. parvum* infected mice treated with ZnO-NPs. G5. *C. parvum* infected mice treated with NTZ loaded on ZnO-NPs. G6.* C. parvum* infected mice treated with *A. sativum* loaded on ZnO-NPs. Triple asterisks indicated a statistical significance at *P* < 0.001 (n = 5).

On day 21 PI, oocyst shedding was quantified and estimated per gram of faeces. The data was analyzed by one-way analysis of variance (ANOVA) with Duncan post-hoc test to show the difference between the means of either infected versus non-infected or versus treated groups. In compared to the infected non-treated control group (G2), oocyst output was significantly reduced in the groups treated with NTZ and *A. sativum* loaded ZnO-NPs and ZnO-NPs alone (*P* ≤ 0.001). G3 treated with NTZ had the largest reduction in Cryptosporidium oocyst shedding (81.5%), followed by G6 (71.1%) treated with *A. sativum* loaded ZnO-NPs, G5 (69.6%) NTZ loaded ZnO-NPs, and G4 (64.3%) treated with ZnO-NPs. However, there was no statistically significant difference between the NTZ loaded ZnO-NPs, NTZ, and ZnO-NPs treated groups (*P* ˃ 0.05). When compared to ZnO-NPs, *A. sativum* loaded ZnO-NPs significantly reduced the oocyst count (*P* ≤ 0.05) (Fig. 9).

### Histopathological examination results

#### Intestine

**Figure 10:**
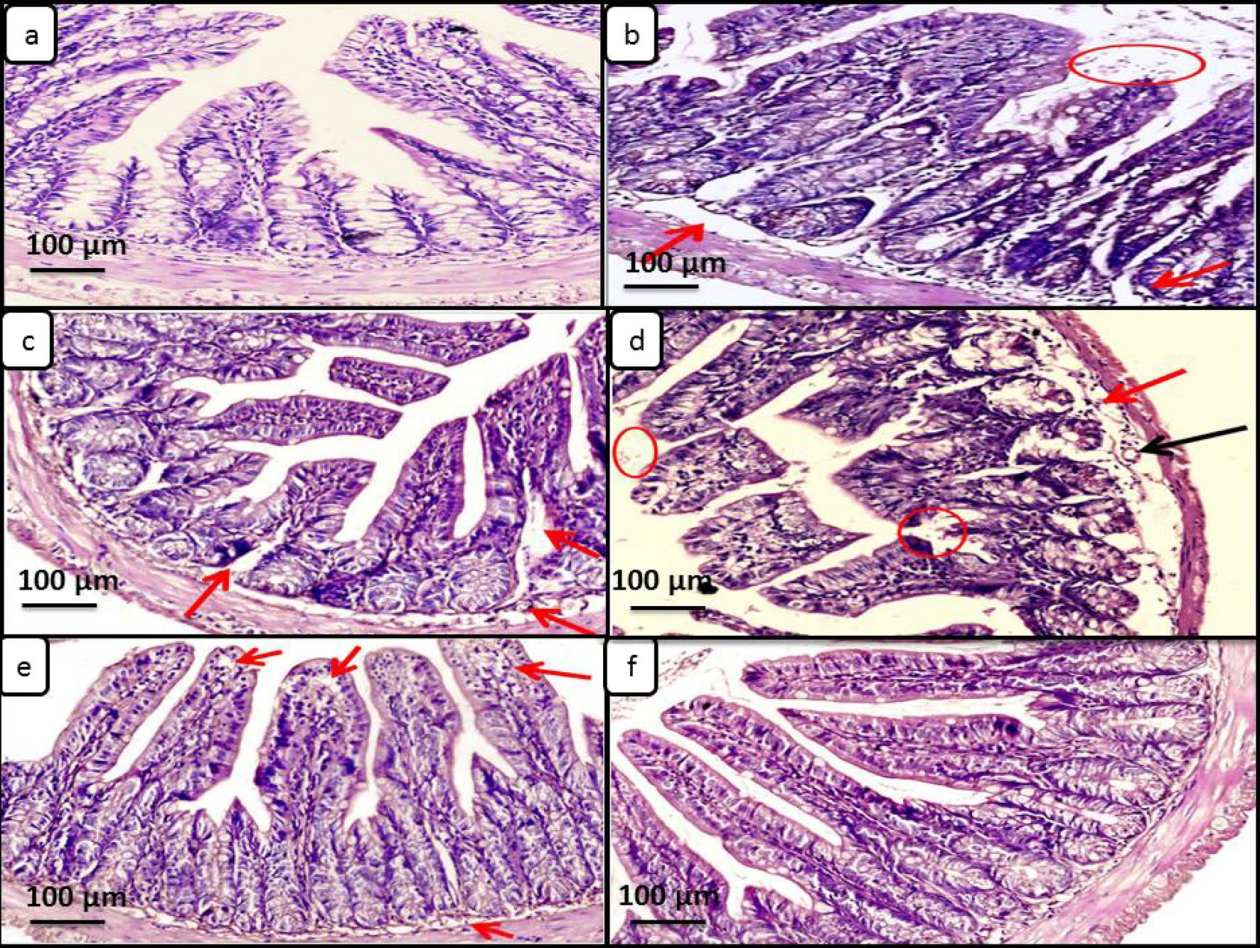
Histopathological ilea sections of different treatment groups stained with hematoxylin and eosin (×100). (**a**) Light microscopy of small intestinal section from negative control group showed normal mucosa; (**b**) Positive control group in which the intestinal lumen showed heavy infestations of oocysts (red cycle) associated with moderate edema (red arrow); (**c**) NTZ treated group showed broadening of villi by moderate inflammatory edema (red arrows) & intestinal villi with absence of *Cryptosporidium* oocysts; (**d**) ZnO-NPs treated group showed minimal residual intraluminal *Cryptosporidium* oocysts (red cycles) associated with marked few inflammatory cells & edema (red arrow), with mild congestion (black arrow); (**e**) NTZ loaded ZnO-NPs treated group showed broadening of villi with moderate inflammatory edema and few inflammatory cells (red arrows), intestinal villi with absence of *Cryptosporidium* oocysts; (**e**) *A.sativum* loaded ZnO-NPs treated group showed almost normal intestinal villi with absence of *Cryptosporidium* oocysts.

Figure 10 illustrates the histopathological examination results of positive control group (G2) showed heavy infestations of the intestinal lumen by oocysts associated with moderate edema and mild inflammatory cells infiltrate as well as congestion of submucosal capillaries. NTZ treated group (G3) showed broadening of villi by moderate inflammatory edema & intestinal villi with absence of *Cryptosporidium* oocysts., ZnO-NPs treated group (G4) showed minimal residual intraluminal *Cryptosporidium* oocysts associated with marked few inflammatory cells & edema with mild congestion. NTZ loaded ZnO-NPs group (G5) showed broadening of villi with moderate inflammatory edema and few inflammatory cells, intestinal villi with absence of* Cryptosporidium* oocysts. *A.sativum* loaded ZnO-NPs treated group (G6) showed almost normal intestinal villi with absence of *Cryptosporidium* oocysts.

#### Liver

**Figure 11:**
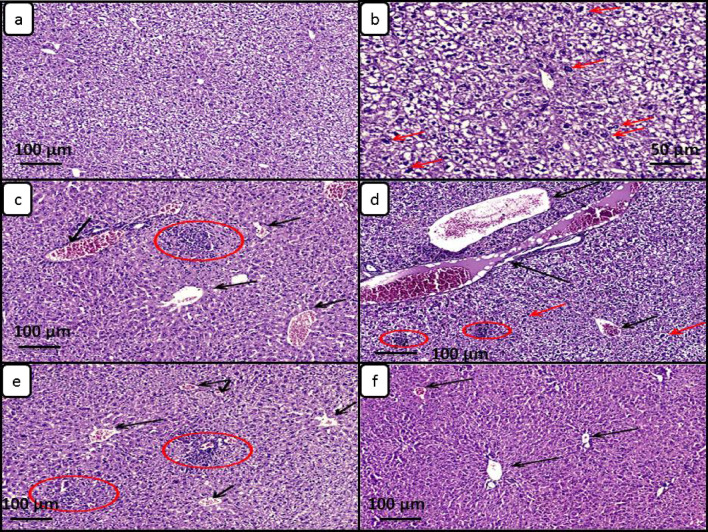
Light microscopy of liver sections from different mice groups included in the study stained with hematoxylin and eosin (× 100). (**a**) Negative control group showed diffuse moderate hydropic degeneration of hepatocytes with vacuolated cytoplasm associated with minimal congestion and absence of inflammation; (**b**) Positive control group showed diffuse hydropic degeneration of hepatocytes which had vacuolated cytoplasm associated with numerous dysplastic cells with hyperchromatic large nuclei (red arrows) (H&E stain × 200); (**c**) NTZ treated group showed mild hydropic degeneration of hepatocytes which had vacuolated cytoplasm associated with marked congestion (black arrows) and moderate lobular chronic inflammation (red circle); (**d**) ZnO-NPs treated group showed marked hydropic degeneration of hepatocytes which had vacuolated cytoplasm associated with marked congestion (black arrows) and mild lobular chronic inflammation (red circles) as well as scattered dysplastic cells with hyperchromatic large nuclei (red arrows); (**e**) NTZ loaded ZnO-NPs group showed mild hydropic degeneration of hepatocytes which had vacuolated cytoplasm associated with moderate congestion (black arrows) and moderate lobular chronic inflammation (red circles); (**f**) *A. sativum* loaded ZnO-NPs treated group showed mild cloudy degeneration of hepatocytes with mild congestion (black arrows) and without inflammation.

Histopathological examination results of liver sections are illustrated in Fig. 11 and Table 1. Positive control group (G2) showed diffuse hydropic degeneration of hepatocytes which had vacuolated cytoplasm and associated with numerous dysplastic cells with hyperchromatic large, (c) NTZ treated group (G3) showed mild hydropic degeneration of hepatocytes which had vacuolated cytoplasm and associated with marked congestion and moderate lobular chronic inflammation, ZnO-NPs treated group (G4) showed marked hydropic degeneration of hepatocytes which had vacuolated cytoplasm associated with marked and mild lobular chronic inflammation as well as scattered dysplastic cells with hyperchromatic large nuclei, NTZ loaded ZnO-NPs (G5) group showed mild hydropic degeneration of hepatocytes which had vacuolated cytoplasm associated with moderate congestion and moderate lobular chronic inflammation, A.sativum loaded ZnO-NPs treated group (G6) showed mild cloudy degeneration of hepatocytes with mild congestion and without inflammation.

**Table 1:** Variable degrees of hepatic injury in different treatment groups included in the study.

Different groups	Degree of hydropic degeneration	Dysplastic hepatocytes	Inflammation	Congestion
Negative Control group	mild Score 1+	Absent	Absent Score 0	Minimal Score 1+
Positive Control group	Marked Score 3+	Few	Absent Score 0	Moderate Score 2+
NTZ treated group	Mild Score 1+	Absent	Marked Score 3+	Moderate Score 2+
Zno-NPs treated group (4)	Marked Score 3+	Few	Marked Score 3+	Mild Score 1+
NTZ loaded on Zno-NPs treated group	Mild Score 1+	Absent	Moderate Score 2+	Moderate Score 2+
*A.sativum* loaded on Zno- NPs treated group	Minimal Score 1+	Absent	Absent Score 0	Moderate Score 2+

#### Lung

**Figure 12:**
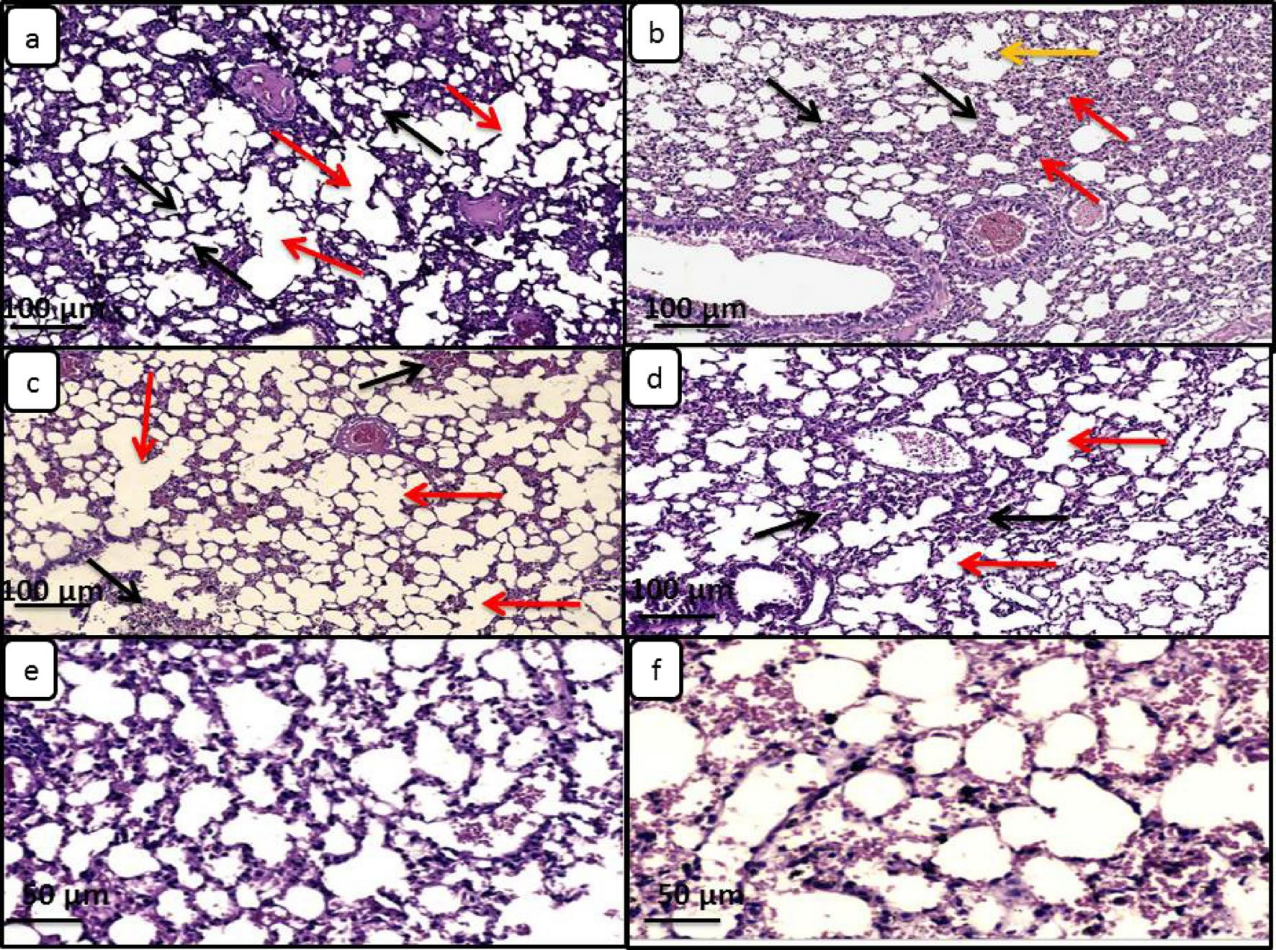
Light microscopy of lung tissue sections from different mice groups included in the study stained with hematoxylin and eosin (× 100). (**a**) Negative control group showed delicate and thin alveolar walls (black arrows) and emphysematous changes with severe destruction of alveolar septa (red arrows); (**b**) Positive control group showed destructed and dilated airspaces and rupture of alveolar septa(yellow arrow), with severe inflammation (red arrows) associated with thickening of some alveolar interstitial spaces (black arrows); (**c**) NTZ treated group showed rupture of many alveolar septa (red arrows) and mild inflammation beside the alveolar spaces (black arrows); (**d**) ZnO-NPs treated group showed moderate inflammation (black arrows) associated with destruction of some alveolar septa (red arrows); (**e**, **f**) NTZ loaded ZnO-NPs and *A.sativum* loaded ZnO-NPs groups showed mild to moderate inflammation between nearly normal alveolar spaces with minimal destruction of alveolar septa in group f.

Lung sections from infected immunosuppressed mice showed moderate pathological lesions such as thick interstitial space, emphysematous changes, destruction of alveolar septa and inflammatory infiltration. These lesions were improved with drug administration as shown in Fig. 12

### Effect of NTZ and different NPs treatment regimens on oxidative parameters from ilea tissue

*Cryptosporidium* infection revealed a significant decline in the level of GSH (*P* ≤ 0.001) compared to negative control groups. In versus to positive control, all treated groups showed a significant increase in GSH levels (*P* ≤ 0.001). Loading NTZ into ZnO-NPs increased NTZ effectiveness and resulted in a considerable increase in GSH level (*P* ≤ 0.001). Furthermore, *A. sativum* loaded ZnO-NPs demonstrated a substantial increase in GSH values when compared to the ZnO-NPs treated group (*P* ≤ 0.001).

**Figure 13:**
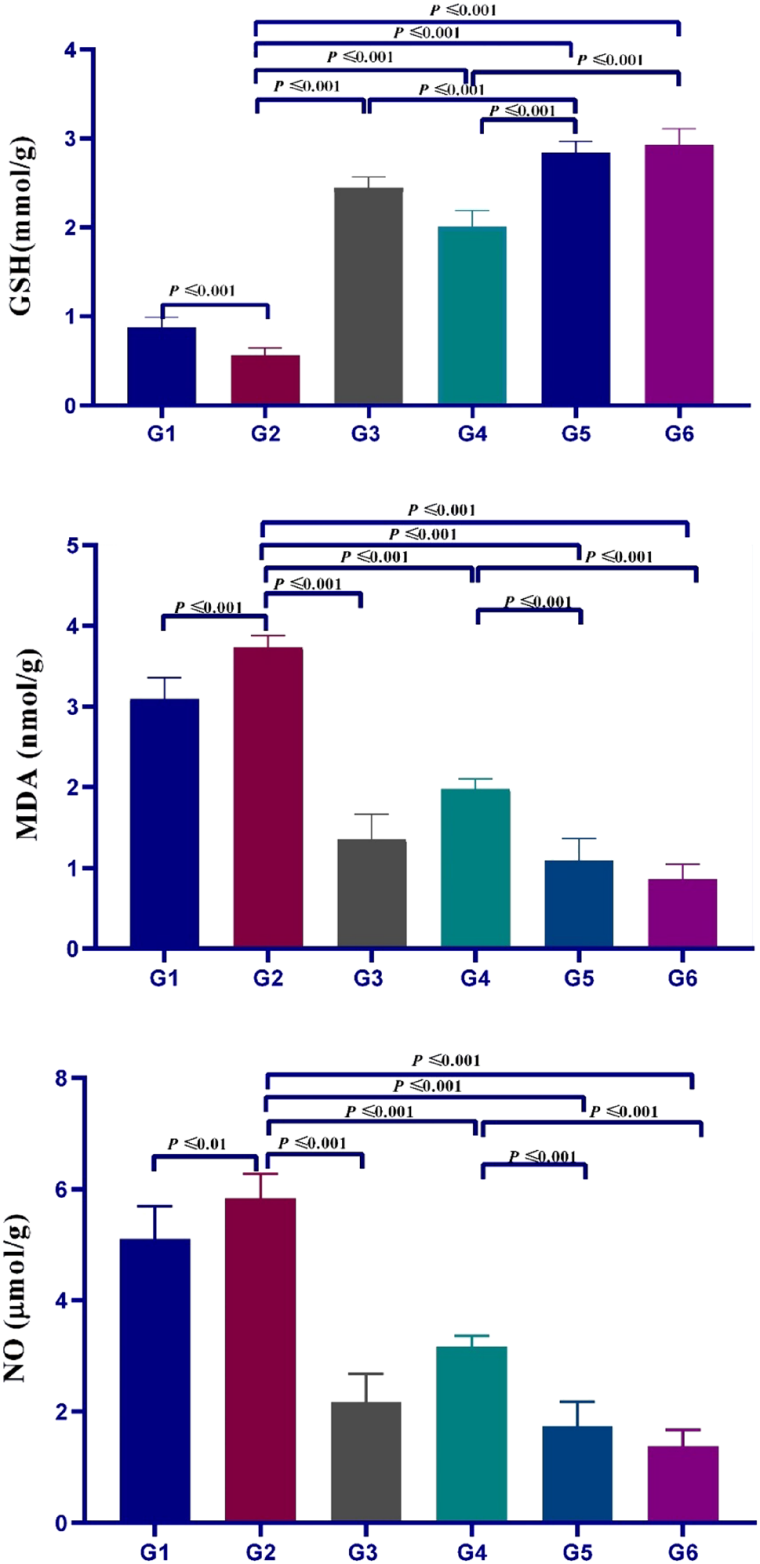
Effect of variable treatment regimens used in the study on GSH, MDA, and NO levels in ileum of infected mice with *Cryptosporidium*. Values were enumerated as mean ± SEM (n = 5).

Moreover, the infection provoked a significant increase (*P* ≤ 0.001, *P* ≤ 0.01, respectively) in the levels of MDA and NO. All treated groups highly ameliorated these changes in MDA and NO compared with negative and positive control groups (*P* ≤ 0.001) with more efficacy to* A. sativum* loaded ZnO-NPs and NTZ loaded ZnO-NPs when compared to ZnO-NPs alone (*P* ≤ 0.001) (Fig. 13).

## Discussion

*Cryptosporidium* spp. is one of the major zoonotic health problems in humans, domesticated animals, and immunosuppressed individuals as well. Despite the rapid development of variable aspects of this protozoan, there has been no effective treatment available until now^75–77^.

Only NTZ is currently approved by the FDA for cryptosporidiosis treatment. Its main mechanism of action depends on inhibition of the basic enzyme in the anaerobic metabolism pathway, pyruvate ferredoxin oxidoreductase^78^. However, it has limited cure rates, with the marked disadvantage of being unbeneficial to immunosuppressed patients and malnourished children^79^.

Targeting and designing new nanoparticle dependent drug carriers achieves enhancement of treatment efficacy^80^. Concerning the characterization results, the SEM images showed the morphological studies for all samples to explain the performance of these materials for biological activity. Their morphology had an impact on the distribution in the body and diffusion rate, hence affecting the pharmacokinetics of its payload^81^. In case of (ZN /All) ZnO appeared as scattered particles filled the whole *A. sativum *surface and that is favored in penetration the wall of parasite. For SEM of Zn/NTZ, the surface appeared as wrapped layers which enhanced its surface area and improved its activity. Significant reduction was observed in the zeta potential value after loading with *A. sativum,* and NTZ. This revealed that interactions between ZnO-NPs with *A. sativum* and NTZ affected the structure and charge of these nanocarriers^82^.

The distribution of loaded nanoparticles inside the parasite matrix can be improved with an increase in the negative surface charge in the intestine. Hence, Zn/All has higher biological activity against parasites than Zn/NTZ and pure ZnO-NPS. The distribution and transport characteristics of a nanoparticle in vivo are significantly influenced by its size. Different-sized nanoparticles in this study might be transported differently within the body and, as a result, suppress parasite growth in different ways.

The most popular scaling method used in pharmaceutical technology since the advent of NEP is dynamic light scattering (DLS). Controlling the size of nanoparticles is a promising technique to target the distribution where the parasites dwell and improve antiparasitic drug residence^83^.

The hydrodynamic diameter of the synthesized ZnO-NPs and the loaded sample with *A. sativum* and NTZ was measured to be 304 nm and 535, 1268, 2726 nm, respectively. When the particle size of loaded samples increases, it is considered evidence of loading-driven particle agglomeration.

In the preceding literature, induction of immunosuppression in mice was done by using dexamethasone, as it was previously used in different studies as a well-known immunosuppressive drug, particularly in mice^41, 84^. It has an inhibitory mechanism on the pathway of interferon-gamma and hence the immune response due to the high activity of glucocorticoids^84^.

In this study, immunosuppressed groups developed skin manifestations after 2 weeks of experimentation. Multiple dermatological lesions were related to the administration of glucocorticoids^41, 85^. Uner et al. noticed petechial haemorrhage in the skin on the mice ears also after 14 days of immunosuppression^86^.

As regards the obtained parasitological results in the present study, *Cryptosporidium* oocyst shedding revealed higher significance in the feces of the infected immunosuppressed non-treated mice group than other infected treated groups all through the period of the experiment. *Cryptosporidium* oocysts occurred in the stool of immunosuppressed mice on the 2nd day of PI with a high percentage, and this is in line with other results^41, 87^. Abdou et al. reported that oocyst shedding continued until the 30th day of PI in experimental *C. parvum-infected* mice^88^.

In the preceding literature, the highest reduction percent of *Cryptosporidium* oocyst shedding per gram feces (81.5%) was detected in G3 (NTZ treated group), followed by ZNO-NPS, *A. sativum-*, loaded ZnO-NPS-treated groups, and the least was found in the NTZ-loaded ZnO-NPS-treated group (G5) (69.6%). The obtained results were in line with some authors^89^ whose reduction percentage of oocyst for the group treated by NTZ was 92.05%, revealing the highest result as well. This was in contrast to Moawad et al.^41^, who found that treatment with NTZ-loaded Chitosan NPs produced the best oocyst counts reduction, followed by the NTZ and Chitosan NPs treatment groups. Mohi-Eldin et al. had pointed out that the reduction rate in oocyst per gram of feces in *Eimeria stiedae* infected rabbits treated with A.sativum loaded ZnO-NPs was less than group treated by A.sativum extract alone^49^.

On the other hand, Dkhil et al. documented that oral administration of 10 mg/kg/day ZNO-NPs for continuous 5 days reduced oocyst shedding and jejunal inflammatory injury significantly in *Eimeria papillate-*infected mice^50^.

In regard to our histopathological examination results of the intestinal sections, the intestinal lumen in the positive control group showed heavy infestations of oocysts associated with moderate oedema and mild inflammatory cell infiltrate, as well as congestion of submucosal capillaries. Previous studies were in agreement with these results^5, 41, 90–92^. They illustrated that infection with *Cryptosporidium* in mice caused some alterations in the intestinal mucosa, including variable degrees of villous atrophy and crypt hyperplasia. Marked oedema in lamina propria was exhibited, and moderate chronic inflammatory cell infiltrates accompanied by congestion with dysplastic changes of the intestinal tract were noticed as well.

Infected mice treated with NTZ alone revealed partial enhancement in the intestinal pathological lesions after being infected with *Cryptosporidium*. This obtained result was in line with Moawad et al. and Abd El Wahab et al. who reported that NTZ-treated rats showed moderate changes when compared to a positive control group that showed severe pathological alterations^5, 41^. Also, the ZnO-NPs-treated group showed moderate improvement in the intestinal lesions.

In the current study, mice received *A. sativum-*loaded ZnO-NPs, followed by NTZ-loaded ZnO-NPs, revealed the highest enhancement in histopathological intestinal lesions. That was indicated by normal intestinal villi and broadening of villi with moderate inflammatory oedema and few inflammatory cells, respectively, with the absence of *Cryptosporidium* oocysts in the intestinal villi in both groups. These obtained findings were in parallel with others who recorded amelioration of the intestinal histopathological sections by detecting minimal *Cryptosporidium* oocysts at the intestinal epithelium with mild inflammatory cellular infiltration in mice receiving combined NTZ-artesunate loaded on polymeric nano-fibre and NTZ-loaded Chitosan NPs, respectively^41, 91^.

According to histopathological changes in liver tissue sections, the positive control group showed marked diffuse hydropic degeneration of hepatocytes that had vacuolated cytoplasm and was associated with moderate congestion. Scattered dysplastic cells with hyperchromatic large nuclei and the absence of inflammation had been noticed. Certad et al. and Abdou et al. demonstrated a high grade of hepatic and bile dysplasia in severe immunodeficiency and immunosuppressed mice^88, 93^. Meanwhile, Elmahallawy et al. reported the hepatic injury in the form of high levels of serum liver enzymes^94^. Also, *C. parvum* oocyst induction led to pathological liver tissue changes in the form of hepatocyte degeneration and focal infiltration with mononuclear cells^95^.

Zno-NPs-treated mice group showed nearly no improvement, and in group treated by NTZ, the pathological alterations ranged from marked to moderate. Treated mice receiving *A. sativum-*loaded ZnO-NPs and NTZ-loaded ZnO-NPs revealed the highest amelioration of the liver picture by finding minimal and mild degrees of hydropic degeneration. Moderate congestion and the absence of dysplastic hepatocytes were noticed in both groups. These results went hand in hand with Moawad et al.^41^, who used NTZ loaded Chitosan NPs in *Cryptosporidium* infected immunosuppressed mice, and Mohi-Eldin et al. who found that garlic-loaded ZnO-NPs had anti-parasitic action on hepatic coccidiosis in rabbits^49^.

The pathogenicity of respiratory cryptosporidiosis has not been fully pointed out. Previous research reported that pulmonary cryptosporidiosis may complicate immunosuppressed subjects and even occur in immunocompetent ones^96^. A similar study about *Cryptosporidium* oocysts establishment within the lung tissue was reported recently^97^.

In the present study, lung tissue sections of the immunosuppressed positive control group showed markedly severe inflammation and thick alveolar interstitial spaces with rupture of the alveolar septa. This finding was illustrated by other recent studies^41, 95^. In immunosuppressed mice groups receiving NTZ, Zno-NPs, and NTZ-loaded ZnO-NPs, the pathological changes ranged from mild to moderate inflammation with partial improvement. Similar results were recorded previously^41, 95^. Moreover, Madbouly et al. found interstitial inflammation and pulmonary haemorrhage in the lung tissue of immunosuppressed infected mice, and they got better after combined therapy with NTZ and atorvastatin^98^. For the first time, treated immunosuppressed mice receiving *A. sativum-*loaded ZnO-NPs showed the highest improvement in the lung picture by finding mild inflammation with minimal destruction of the alveolar septa.

*A.sativum* has the ability to treat experimental cryptosporidiosis through disruption of the normal physiological bioactivities of the parasite, such as food absorption, reproduction, and mobility. Immune system enhancement by stimulating the natural killer cells' activity and phagocytosis may be another mechanism^99^.

The antioxidant activity of all garlic compounds is well documented, with allicin and S-allylcysteine showing the highest activity through enhancement of endogenous radical scavenging mechanisms, inhibition of free radical formation, and elevation of the antioxidant cellular enzymes^100^. The antioxidant activity of *A. sativum* and its compounds becomes of great interest as regards their anti-hepatotoxic, anti-atherogenic, and anti-cancer properties^13^.

Furthermore, ZnO-NPs have the ability to prevent the wasting of GSH through oxidative damage prompted by infections, as previously demonstrated by Dkhil et al.^101^. It is supposed that the antibacterial activity of these metal oxide particles is attributed to the generation of active oxygen species^102^. Joe et al. illustrated that ZnO-NPs have the ability to disrupt the oocyst wall glycoproteins, facilitating penetration and consequently increasing harmful Zn2 + ions^103^. In addition, Siddiqi et al. and Prasad et al. observed that when ZnO-NPs were exposed to light, their photosensitive capabilities were activated, resulting in the formation of reactive oxygen radicals that affect the parasite virulence-associated surface glycoprotein molecules with decreased oocyst infectivity^104, 105^. Moreover, the tiny size of the NPs allowed for easy passage through cell membrane pores to exert a direct, harmful effect^106^. Furthermore, it binds to DNA molecules, disrupting critical metabolic processes in *C. parvum*^107^.

In the current study, treatment with *A. sativum* and NTZ-loaded ZnO-NPs groups produced a significant increase in GSH levels compared to non-infected, infected control groups, and NTZ- and ZnO-NPs-treated groups. All treated groups improved changes in NO and MDA values compared with control groups, with more efficacies for *A. sativum-*loaded ZnO-NPs and NTZ-loaded ZnO-NPs when compared to ZnO-NPs alone. These obtained results match with Abdelhamed et al. who stated that the highest free radical scavenging capacity in serum and different tissue homogenates was observed in mice receiving combined NTZ and artesunate-loaded polymeric nanofiber^91^, and Dkhil et al. who concluded that administration of ZnO-NPs helped in protection against the oxidative stress negative effects in *E. papillata-*infected mice^50^. Moreover, ZnO-based nano-formulations inhibited *Leishmania tropica* promastigotes growth because of the production of reactive oxygen species by these NPs^108^.

The antioxidant property of A. sativum is owed to many bioactive compounds that act as antioxidants either directly through modifications in the pre-apoptotic oxidative mechanism or indirectly via stimulation of the endogenous synthesis of antioxidant enzymes or molecules. The capability of the antioxidants to scavenge generated free radicals during cell respiration protects the human body against infections^109^.

ZnO-NPs almost show their antimicrobial characteristics through two main mechanisms: their direct effect through inhibition of cell growth, impairment of cell permeability, and apoptosis induction. The second is their indirect effect by H_2_O_2_ production that induces oxidative stress and the release of zinc ions in the environment, which enables their cell wall penetration and their antimicrobial effects^110, 111^.

Our results demonstrated that ZnO nanomaterials can be used further in a wide range of biological applications, and this is equally important, as reported by Khan et al.^112^. The obtained results support our hypothesis that ZnO-NPs could have significant medical importance in pharmaceutical and medical science for their bio-catalytic activities and improve their utilisation in different biological antiparasitic applications. The therapeutic properties of garlic were empowered by being loaded with ZnO-NPs and acting synergistically, causing potent anti-inflammatory and anti-oxidative effects.

## Conclusion

In the current study, the synthesis of low-cost ZnO-NPs through co-precipitation was investigated. These nanoparticles were modified to be nanocarriers for A. *sativum* and NTZ. These newly materials were well characterized and for the first time ZnO-NPs were used as a successful, effective alternative therapy in treating experimental cryptosporidiosis especially when combined with other treatments that enhanced their antioxidant activity. The study also provides an economical and environment-friendly approach towards the synthesis of novel delivery for antiparasitic applications. Unlikely, this study didn’t investigate the effect of ZnO-NPs loaded materials in vitro or evaluate their prophylactic effect on *C. parvum* infection. To obtain a dosage response curve for the used drugs, more doses should be further evaluated. Additional metal oxides or new therapeutic measures could be used to treat parasitic infections and manage a dose-effective regimen that protects against any hazardous side effects that might occur over an extended period of time.

## Data Availability

The data that supports the findings of this study are available in the material of this article.
